# Strategies for Improving CAR T Cell Persistence in Solid Tumors

**DOI:** 10.3390/cancers16162858

**Published:** 2024-08-16

**Authors:** Megen C. Wittling, Anna C. Cole, Brianna Brammer, Kailey G. Diatikar, Nicole C. Schmitt, Chrystal M. Paulos

**Affiliations:** 1 Department of Surgery/Oncology, Winship Cancer Institute, Emory University, Atlanta, GA 30322, USA; 2 Department of Microbiology and Immunology, Emory University, Atlanta, GA 30322, USA; 3 School of Medicine, Emory University, Atlanta, GA 30322, USA; 4 Department of Otolaryngology, Emory University, Atlanta, GA 30322, USA

**Keywords:** CAR T cells, adoptive cellular therapy, solid tumors, persistence

## Abstract

**Simple Summary:**

CAR T cell therapies have been successful at treating patients with blood cancers and are now FDA-approved therapies for various types of leukemias and lymphomas. However, there are many challenges to using CAR T cells to treat patients with solid tumors. In fact, no CAR T cell product is currently FDA-approved in this context. Optimism remains for this promising therapy to treat solid tumors in the future. Many different strategies are being employed to improve the efficacy of next-generation T cell products. This review focuses specifically on one key problem with current CAR T cell products for solid tumors: persistence. We describe the emerging strategies being used to improve this element of CAR T cell therapy.

**Abstract:**

CAR T cells require optimization to be effective in patients with solid tumors. There are many barriers affecting their ability to succeed. One barrier is persistence, as to achieve an optimal antitumor response, infused CAR T cells must engraft and persist. This singular variable is impacted by a multitude of factors—the CAR T cell design, lymphodepletion regimen used, expansion method to generate the T cell product, and more. Additionally, external agents can be utilized to augment CAR T cells, such as the addition of novel cytokines, pharmaceutical drugs that bolster memory formation, or other agents during either the ex vivo expansion process or after CAR T cell infusion to support them in the oppressive tumor microenvironment. This review highlights many strategies being used to optimize T cell persistence as well as future directions for improving the persistence of infused cells.

## 1. Introduction

Adoptive cellular transfer (ACT) therapy encompasses tumor-infiltrating lymphocyte (TIL) therapies as well as those that involve engineering T cells with either a T cell receptor (TCR) or chimeric antigen receptor (CAR). With TIL therapy, T cells from the tumor are expanded to large numbers before infusing them back into patients, whereas with both CAR and TCR-based therapies, the patient’s circulating T cells are obtained via leukapheresis and engineered with a receptor that can recognize a target expressed by the cancer [1]. CAR T cell therapy is particularly exciting and is decades in the making, with CAR T cells first described in the 1980s [2,3,4]. However, it was not until 2017 that the first CAR T cell therapy was FDA-approved—targeting CD19 on malignant B cells for use in treating patients with acute lymphoblastic leukemia (ALL) [5].

Since then, several additional CAR T cell therapies have been approved to treat patients with hematological malignancies, namely targeting CD19 and BCMA [6]. Current FDA-approved T cell therapies are listed in Table 1. This includes CAR T cell products for use in hematologic malignancies as well as the newly approved TIL product lifileucel and the TCR product afamitresgene autoleucel for use in melanoma and sarcoma, respectively. The TIL product lifileucel was approved in February 2024 for use in patients with melanoma unresponsive or refractory to immune checkpoint blockade therapy and was the first T cell therapy approved for use in solid tumors. Less than 6 months later, in August 2024, the second T cell therapy for solid tumors was approved for patients with unresectable or metastatic synovial sarcoma that have received chemotherapy previously, express the TCR-target antigen MAG121E-A4, and have one of the following HLA antigens: A*02:01P, -A*02:02P, -A*02:03P, or -A*02:06P [7]. While CAR T cell therapies are not yet approved for use in patients diagnosed with solid tumors, these rapid developments in this space highlight the potential future success of both CAR T and other T cell therapies in this context.

As demonstrated in Table 1, the approved CAR T cell products are for use in treating hematologic malignancies. Four products (Breyanzi, Kymriah, Tecartus, and Yescarta) target CD19, a cell surface marker expressed ubiquitously on B cells throughout development. Targeting CD19 via a CAR enables T cell products to more effectively treat B cell malignancies, directly recognizing a target expressed on malignant cells [8,9]. Abecma and Carvykti are additional CAR T cell products approved for the treatment of multiple myeloma, targeting the B cell maturation antigen (BCMA) expressed on malignant plasma cells in this cancer [10]. In contrast, many CAR T cell products designed for solid tumors target molecules that are expressed in both malignant and healthy tissues, increasing the risk of off-target effects, including organ damage. Additionally, a key difficulty in CAR T cell therapy for solid tumors is the necessity for CAR T cells to traffic into the site of the malignancies. This barrier is reduced when using CAR T cell therapies for hematologic malignancies, as they are able to effectively interact with their target cells while in circulation.

Both clinical and preclinical research suggests CAR T cells need to be further optimized to make an impact against solid tumors. Previous work has uncovered many reasons why CAR T cell therapy remains challenging for use in solid tumors, including (1) difficulty trafficking to tumors, (2) tumor heterogeneity, (3) T cell exhaustion, (4) decreased T cell persistence, (5) metabolic stress, (6) immunosuppressive tumor microenvironments, and (7) on-target off-tumor toxicities, among several other barriers reviewed elsewhere [11,12,13,14,15]. This new review focuses specifically on one of these barriers: persistence. Persistence describes the ability of T cells to survive long-term after infusion into patients and is associated with improved survival in hematological malignancies and importantly, in solid tumors [16,17]. Yet CAR T cell persistence in the context of solid tumors remains challenging, as they do not immediately encounter their CAR target and instead require time to traffic to the malignancy, during which they can encounter many stressors. Therefore, it is of utmost importance to improve CAR T cell persistence in patients with solid tumors.

**Figure 1:**
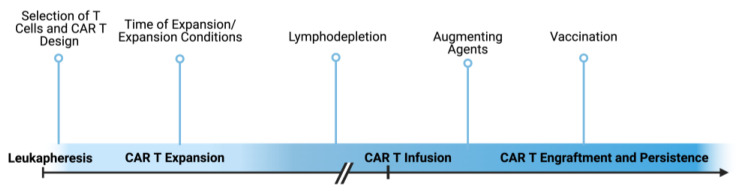
Intervention strategies to enhance CAR T cell persistence. Depicted is the general timeline for CAR T cell treatment as well as points indicated at which the persistence of CAR T cells can be impacted. Made using BioRender.

This review seeks to define the plethora of ways in which translational scientists can enhance persistence as a means of improving CAR T cell efficacy in treating solid tumors. There are numerous points during the CAR T cell manufacturing process and after infusion into the patients, as displayed in Figure 1, that can be manipulated to augment persistence. This includes the initial design of the CAR T cell—such as what types of costimulatory domains are incorporated, the receptor affinity, and whether the CAR T cell has been engineered with additional attributes that will impact its ability to engraft and persist. Secondly, the conditions for expansion and level of stemness of the CAR T cell product are important contributors to persistence. Before patients receive these T cell products, they can undergo lymphodepleting regimens that greatly impact the ability of CAR T cells to engraft and persist. Lastly, after CAR T cell infusion into patients, additional agents or vaccination strategies can improve the persistence of adoptively transferred cells. These factors are discussed herein.

## 2. Discussion

### 2.1. CAR T Cell Design

The design of the CAR T cell construct is an important aspect to consider. CAR T cells are normally designed with an extracellular single-chain variable fragment (scFv), followed by the transmembrane domain, an intracellular region that contains the CD3ζ activation domain, and costimulatory domains [18]. These signaling elements differ depending on the generation of the CAR T cell, with the first generation only containing the CD3ζ domain, while the second and third generations have one or two costimulatory domains, respectively [18].

Depending on which costimulatory domain(s) are incorporated, CAR T cells can be endowed with different properties. Current FDA-approved CAR T cell therapies use either CD28 or 4-1BB (CD137) costimulatory domains. Canonically, CD28 is more associated with effector phenotypes, while 4-1BB is associated with enhanced persistence due to their different kinetics, although both have demonstrated efficacy in preclinical models and clinically in humans [19,20,21]. Additionally, when combined in a third-generation CAR, these two costimulatory domains led to more robust persistence in leukemia and solid tumor models [19,22]. Multiple third-generation CAR T cell products are in early-phase clinical trials, spanning the treatment of patients with thyroid cancer, neuroblastoma, and melanoma. Treatment with these products shows great promise, demonstrating CAR T persistence and disease response [23,24].

**Figure 2:**
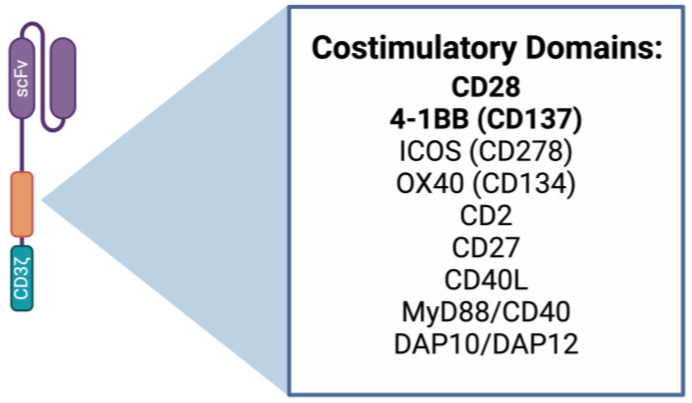
Costimulatory domain possibilities in CAR constructs. Depicted is a first-generation CAR T cell with a zoomed-in region on the costimulatory domain. Different costimulatory domains being tested in CAR T cell design are listed. Domains in bold are used in current FDA-approved CAR T cell products. Note that these domains can also be combined in tandem with another domain in a third-generation CAR T cell (not depicted). Made using BioRender.

Several costimulatory domains can be incorporated into CAR constructs (Figure 2). These cues can be incorporated as the sole costimulatory domain (second-generation CAR) or in combination with additional costimulatory molecules (third-generation CAR). ICOS was reported to improve persistence and, when combined with 4-1BB in a third-generation CAR T construct, mediated superior antitumor effects against lung tumors [25]. CD2 has also been incorporated into MUC1-targeting CAR T constructs in a Phase I clinical trial for solid tumors [26]. Other novel CAR constructs have also been made, integrating MyD88 combined with CD40 to enhance CAR T persistence [27,28] or DNAX-activating proteins (DAP) 10 and 12 [29,30,31]. CD27 inclusion in CAR constructs also demonstrates potential benefit to CAR T survival [32]. 

There remains a question of which of these costimulatory domains and in what combination in the construct will lead to the best CAR T cell products for solid tumors. This uncertainty is in part due to making T cells with the “right” biology. In other words, while it is well appreciated that effector functions are important in CAR T success, the sustained signaling and activation that foster these properties can also lead to their dysfunction [33]. Sustained activation even in the absence of antigen stimulation is also defined as tonic signaling and can limit antitumor efficacy [33]. 

Strategies to ameliorate tonic signaling have been reviewed elsewhere [34] and are an important consideration when designing CAR constructs. Key factors influencing tonic signaling include the folding and stability of the single-chain variable fragment (scFv), spacer flexibility and length, target antigen affinity, and the choice of transmembrane and costimulatory domains [34]. DAP12 can be incorporated as a costimulatory domain to reduce tonic signaling due to its immunoreceptor tyrosine-based inhibitory motif (ITIM) [29]. The choice of scFv in CAR T cells is also crucial for success, as its stability can lead to self-aggregation and heightened tonic signaling [35]. Different “tonic signaling indices” for commonly used CARs were calculated using normalized CD69 levels [36]. This same group found variable levels of tonic signaling depending on the construct and that high levels of tonic signaling correlated with increased T cell exhaustion signatures [36]. As a result, several strategies are being employed to aid in tonic signaling effects, which are reviewed elsewhere [34]. 

Logic-gated strategies and dual-targeting CAR T cells offer exciting solutions for minimizing tonic signaling and off-target effects. Boolean logic gates—“AND,” “NOT,” and “OR”—enable CAR T cells to activate only in the presence of specific antigen combinations (AND), reduce activation in non-target cells (NOT), or respond to any of multiple molecules (OR) [37]. One unique logic-gated strategy involves using combinatorial antigen-sensing circuits, specifically SynNotch circuits. SynNotch receptors are designed so that an extracellular cue, such as initial binding to tumor antigen, can then trigger an engineered cellular response, such as the expression of an additional CAR [38]. In CAR T cells, this feature enables more precise CAR expression at tumor sites and reduces the tonic signaling typically observed with CAR products [38,39], allowing for localized CAR expression and reducing on-target, off-tumor effects [39]. SynNotch CAR T cells have displayed both increased persistence and antitumor activity in mice bearing solid tumors and represent a promising therapeutic strategy [40]. 

Herein, we review changes to CAR design that can augment their persistence in vivo; however, it is important to acknowledge that a plethora of other elements can be considered for novel CAR design to generally improve their efficacy in solid tumors. Some of these areas of ongoing research pertain to choosing the optimal target antigen, minimizing toxicities and off-target effects, and considering synapse formation and temporal dynamics of CAR signaling [18]. Furthermore, spacer length and receptor affinity are crucial elements known to impact CAR T cell efficacy [41]. Additionally, to combat novel barriers such as immunosuppressive microenvironments and trafficking through tissue, there may be other elements that need to be incorporated into CAR designs for therapy success in patients with solid tumors.

### 2.2. Genetic Alteration of CAR T Cells

The gene expression of the CAR-modified T cells can also be modulated to impact CAR therapy efficacy and persistence. This is primarily accomplished in two ways: overexpressing pathways to promote CAR T cell fitness or inactivating pathways that are deleterious for T cell therapy efficacy. Many genes have improved CAR T cell products when overexpressed. One recent strategy involved overexpressing the active form of the transcription factor FOXO1 (FOXO1-ADA) in CAR T cells, finding that when increased, CAR T cells had stemness and improved antitumor abilities in mice with solid tumors [42]. 

Similar strategies have been used to overexpress other important molecules in T cell fitness. Overexpression of Ado deaminase (ADA-OE), an enzyme important in metabolizing the immunosuppressive metabolite Ado, led to increased properties of stemness [43] and could be utilized in solid tumor models in the future. When overexpressed, c-Jun led to increased CAR T cell expansion as well as enhanced functionality in murine solid tumor models [44]. Metabolic mediators like GLUT1 and GSNOR have also been found to enhance CAR products when increased [45,46]. Persistence, specifically, was found to be improved by overexpression of the anti-apoptotic BCL-XL [47]. Chemokines can also be overexpressed to mediate the improved effects of CAR therapies [48]. There are a plethora of genes that could ultimately be upregulated in CAR T cells to mediate improved responses, and this strategy is yet another means to improve CAR therapies.

One strategy used to identify genes that may impact stemness was to screen for mutations found in T cell neoplasms associated with improved T cell fitness [49]. The effect of these mutations on CAR T cell phenotype and persistence was then assessed using pooled screening tools and a xenograft model. Specifically, CAR T cell persistence was increased when genes such as MYCN, CARD11/PIK3R3 fusion, CCND3, STAT3, and others were enriched [49]. CARD11–PIK3R3 was further investigated and found to improve CAR T cell efficacy in multiple solid tumor models as well as enhance their survival [49]. Tools such as this screening model can be used to help identify targets beneficial to T cell survival that can then be overexpressed to increase CAR T survival.

Conversely, inactivation of various pathways or enzymes can also enhance CAR T cell products. For example, groups have found that disruption of SUV39H1, a histone methyltransferase important in Th2 commitment [50], improves CAR T cell persistence and endows them with a more “stem-like phenotype” in both leukemia and solid tumor models [51,52]. PD-1, a known mediator of T cell inhibition via the PD1-PDL1 axis [53], has also been knocked out in CAR T cells both in preclinical models and clinical trials for solid tumors [54,55,56,57]. CRISPR/Cas9 has revolutionized our ability to modulate CAR therapies for patients [58], and its use to knockout other inhibitory molecules like CTLA-4 [59], Lag-3 [60], and other factors can improve current therapies. Ultimately, perturbing gene expression in CAR T cell products is a known strategy that can enhance the persistence and fitness of cells. This strategy is one to consider when optimizing their use for solid tumors.

### 2.3. Increasing T Cell Stemness

T cell stemness is an important aspect to consider for ACT therapy, as the infusion of more “memory-like” CD8 T cells has been correlated with improved persistence [61,62]. Decreased differentiation of T cells improves antitumor responses in solid tumors [63], and enhancing this attribute has been discussed as a major means to potentiate cellular therapy [64,65]. However, there does remain a balance between maintaining stemness and harnessing the cytolytic activity of T cells. There are many ways to increase stemness, including the initial selection of cells for CAR T cell transduction, the addition of cytokines, and drugs that can be added to culture during expansion.

Selecting T cells with naïve or stem-like properties can produce superior ACT products with distinct advantages in preclinical models [66,67]. Naïve CD8 T cells can be selected from bulk lymphocytes for transduction as one means to enhance CAR T therapies [68,69]. Likewise, a subset of CD4 cells, Th17 cells, have stem-like properties [70], and when redirected to express a CAR, they can lead to tumor regression in preclinical models [71,72]. Therefore, expanding cells with these polarizing cytokines or selecting them via extracellular markers is an additional way to improve CAR products. For instance, our team found increased expression of CD26 on CD4 T cells to mark a uniquely effective T cell population with enhanced migration, stemness, and persistence that may be of particular benefit for solid tumors [73,74]. These cells can be selected prior to transduction and expansion and have shown efficacy in CAR T models against hard-to-treat solid tumors [73,74].

Exogenous cytokines also impact T cell phenotypes. While the current most common approach is to expand cells in IL-2 (promoting T cell differentiation [75]), novel approaches using other cytokines have seen positive results [76]. IL-7 and IL-15 increase the survival and stemness of both CD4 and CD8 T cells [77,78,79]. When used during expansion, these cytokines can improve T cell stemness—increasing memory T cell populations that possess beneficial stem-like properties in addition to improved effector function in a model of B cell malignancy [61]. Similar strategies improve CAR T cell efficacy for solid tumors. One group found that by using a Naïve Pan T Cell collection kit (Miltenyi) to select for naïve T cells and subsequently activating them with CD3/28 and expanding with IL-7 and IL-15, there was a marked increase in stemness and improved antitumor activities [68]. Using these cytokines to augment CAR T cell products is a viable strategy to increase the stemness and persistence of ACT products. Additional cytokines can also enhance stemness features, including IL-24 [80] and IL-21 [81,82,83]. These may additionally add benefit to CAR T cells for solid tumors.

Other strategies to enhance stemness include using pharmacologic methods either during the expansion process or after infusion. The PI3-Kinase pathway in particular has been studied for its impact on T cell biology [84,85,86], and blockade of this pathway enhances ACT products [87,88,89]. This is at least in part mediated via increasing transcription factors in the Wnt/β-catenin pathway called Tcf1 and Lef1, associated with T cell stemness [88]. Various PI3K inhibitors have been reported to increase CAR T cell efficacy in leukemia models [90,91] and in TCR-based models for solid tumors [88,92,93,94]. Additional pharmacologic agents such as HDAC inhibitors [95], metformin [96], and mediators of Wnt signaling [97] enhance antitumor activity and may show promise for combining with CAR T cell therapy for solid tumors.

The costimulatory molecules used in T cell expansion and their potency are other variables often altered in the CAR T cell manufacturing process to improve phenotype. Beads or plate-bound antibodies are commonly used to activate T cells during the expansion process [76]. CD3/28 beads are most commonly used, but additional bead products such as CD3/ICOS beads can enhance certain CAR T cell products [71]. Moreover, the bead-to-T cell ratio using this CD3/ICOS strategy impacts CAR T responses as well and is yet another variable to consider [98]. Additional costimulatory molecules (CD137, CD2, CD40, etc.) have also been added to bead products to affect T cell phenotype and persistence, many of which are commercially available [99,100,101]. While the majority of current protocols utilize bead activation, others have used non-activated T cells as well [102].

Notably, other aspects need to be considered for generating the optimal CAR T cell for solid tumors. These factors include the expansion platform and level of oxygenation of the cells during manufacturing [103]. Transduction of the CAR construct is yet another factor that may impact their phenotype, function, and stemness. Modality, reagents used, and multiplicity of infection (MOI) are other factors to consider. The current FDA-approved products use lentiviral and retroviral methods [104]. However, non-viral methods also exist that can be used, such as the transposon systems [105,106,107]. CAR T cells engineered with the piggyBac transposon system have even been utilized in a clinical trial for non-small cell lung cancer patients [108], and this and other strategies may differentially impact T cell phenotype. Manufacturing strategies to improve CAR T success have been described elsewhere [109], encompassing how T cell activation, transduction, expansion, and other elements can impact the fate of CAR T cell products. 

### 2.4. Cytokine Engineering

Many novel strategies to improve CAR T cell persistence involve cytokine engineering. The benefits of cytokines are described above in relation to their ability to enhance T cell stemness, yet there are additional cytokines helpful in augmenting CAR T cell persistence as well as unique strategies to increase the availability and potency of cytokines. One such agent includes rhIL-7-hyFc (efineptakin alfa, NT-I7), an interleukin-7 fusion protein that increases effector memory CAR T populations when injected into mice and improves overall CAR T persistence in mouse models [110].

As described previously, IL-15 can also increase the memory T cell phenotype, and there have been similar studies regarding its ability to benefit CAR T cell persistence. IL-15 can be administered systemically to enhance T cell persistence [111,112]; however, this can be associated with fever and rigors in part due to the marked expansion of natural killer (NK) cells [113]. Therefore, another strategy is to armor CAR T cells to produce cytokines, thereby decreasing systemic effects. CAR T cells engineered to produce membrane-tethered IL-15 alone or in combination with IL-21 led to enhanced persistence and antitumor responses [114,115]. Membrane-bound IL-15 (mbIL15) co-expressed with a second-generation CAR improved long-term engraftment even in the absence of tumor antigen [115]. IL-15 can also modulate the tumor microenvironment (TME) when secreted by CAR T cells to decrease myeloid-derived suppressor cells (MDSCs) in a glioblastoma (GBM) model [99]. These techniques aim to increase CAR T persistence and efficacy through cytokine modulation without the toxicities associated with systemic administration.

Unique strategies for delivering IL-2 via a CAR T cell product have also been investigated. This is particularly important, as systemic administration of IL-2 can lead to some toxicities [116,117,118]. To avoid these side effects, one group designed an orthogonal IL-2 and IL-2Rβ pair mutated in such a way as to prevent binding to wild-type human IL-2 [119]. This approach allowed for CAR T cells to be fitted with orthogonal IL-2Rβ, and upon administration of orthogonal IL-2 to CAR recipients, transferred CAR T cells were more specifically impacted while minimizing the systemic effects of IL-2 on host cells [119].

Beyond homeostatic cytokines, IL-12-secreting CAR T cells also show improvement in antitumor efficacy [120,121]. IL-12 has poor tolerability when administered systemically [122]. To abrogate this, CAR T cells can be “armored” to produce IL-12, allowing for more localized delivery. This strategy led to enhanced expansion and efficacy in mice with ovarian cancer as well as impacted tumor-associated macrophages [123], demonstrating how cytokines can impact the tumor microenvironment. It is likely that tumor microenvironments may respond differently to cytokines released by armored CAR T cells, depending on the malignancy. IL-12 can also be injected intratumorally [124] or as a plasmid/lipopolymer complex [125] for safer delivery. However, scientists have also begun armoring CAR T cells to produce other inflammatory cytokines in the IL-12 family, including IL-23.

IL-23-producing CAR T cells are particularly promising, able to regress neuroblastomas to a greater extent than IL-15- or IL-18-secreting CAR T cells [126]. Moreover, IL-23-producing CAR T cells had a better safety profile based on weight loss as a measurement [126]. Follow-up work, however, has shed a positive light on the impact of IL-18-producing CAR T cells to augment tumor immunity, which inspired a clinical trial testing the impact of IL-18 secretion by anti-CD19 CAR T cells in patients [127]. Along this line, CAR T cells armored to produce IL-18 also demonstrate preclinical benefits in solid tumors [128].

Several research groups have reported how a subset of cytokines, chemokines, can be utilized to enhance T cell homing and responses. Chemokines are directly relevant to CAR T cells, as their expression can mediate immune cell trafficking and their ability to migrate to and then infiltrate the tumor [129,130]. Particularly in the solid tumor context, CAR T cells can have difficulty penetrating the malignant tissue [131]. As different chemokines and chemokine receptors are expressed by tumors and have been correlated with infiltration and survival, this system can be hijacked to modulate CAR T cell homing towards the tumor [130,132,133]. For example, forced expression of CXCR6 improved the migration of CAR T cells into pancreatic tumors expressing CXCL16 in mice [134]. CXCR2-expressing cells also homed better to the tumors [135]. Clinical trials of CAR T cells combined with chemokine-based approaches are underway (NCT03602157, NCT05060796), and the role of these chemokines and chemokine ligands is an important aspect to consider in CAR T success. 

Cytokines can shape both T cells and the surrounding tumor microenvironment. They can be administered systemically or can be produced by the CAR T cells themselves, as in the case of armored CARs. Especially in the context of solid tumors, where T cell survival and trafficking are of immense importance, cytokines play an important role. Cytokines can enhance CAR T cell persistence by promoting survival and growth, and they also play a crucial role in enabling effective migration to tumors. In closing, armoring CAR T cell therapies with distinct cytokines, chemokines, and other survival cues can ultimately improve their persistence, and all of the strategies discussed in this section have the potential to enhance CAR therapies for patients with solid tumors.

### 2.5. Decreased Expansion Time

Another very important aspect to consider is the expansion time of ACT products. CAR T cells are commonly expanded for around 9–14 days prior to infusion into patients, with some variability in this approach [136,137,138,139,140]. However, our lab has found that by decreasing this expansion time, we can deliver a superior ACT product [141]. Additional groups have similarly developed efficacious ACT products in a shorter time frame, finding that non-activated CAR T cells transduced and prepared within even 24 h have the potential to be efficacious for patients [102,142].

Importantly, by decreasing the time of ex vivo expansion, stemness properties can often be sustained or even enhanced, and clinical trials utilizing approaches to decreased expansion have shown promise [143]. CD8 T cell maturation was correlated with the time to expansion in one study [144], with decreased naïve T cells detected as expansion time was increased. However, it should be noted that the expansion platform used to generate ACT products can also affect these T cell phenotypes [103]. It should additionally be appreciated that by decreasing the expansion time, the final yield of CAR T cells may be compromised. Thus, it might be important to increase the number of T cells used during the manufacturing process. The enhanced T cell properties manifested by using a shorter expansion protocol must be balanced with this consideration. Considering these factors, we can optimize CAR T cell conditions for solid tumors and employ strategies to maintain stemness throughout development.

### 2.6. Lymphodepletion

**Figure 3:**
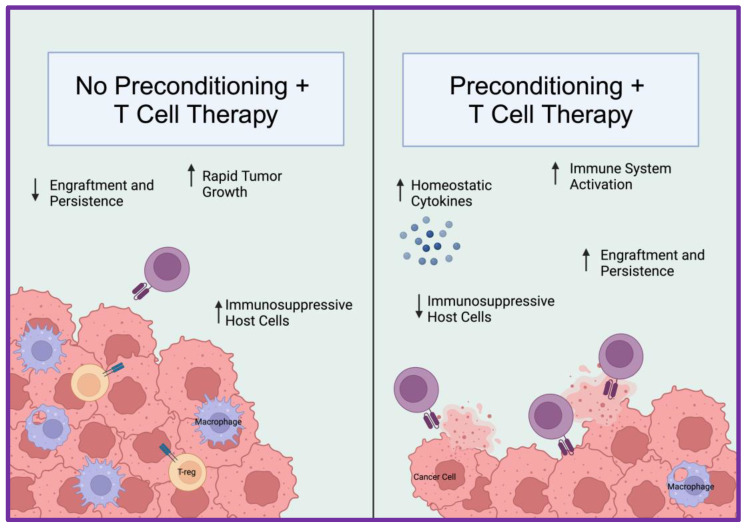
Effects of lymphodepletion. Depicted are several effects of lymphodepletion on adoptive cellular therapy. Made using BioRender.

One strategy to increase CAR T cell persistence is to precondition the patient with lymphodepletion prior to infusing the novel therapeutic product. Lymphodepletion directly augments the engraftment and persistence of transferred CAR T cells and TIL products, thereby improving long-term survival. Lymphodepletion can be mediated in several ways, ranging from treating the individual with radiation to chemotherapy-based regimens, both of which have been reported to improve ACT success in patients [145,146]. There are several mechanisms underlying the effectiveness of lymphodepletion: (1) increasing physiologic space for the transferred cells to thrive; (2) decreasing regulatory cell populations, including regulatory T cells (Tregs) and MDSCs, that blunt the expansion of infused T cells; (3) inducing immune activation of the innate immune system from microbial ligands; and (4) decreasing cytokine sinks, allowing for more available circulation of homeostatic cytokines (such as IL-7 and IL-15) that can directly promote the expansion and function of the transferred CAR T cells [146,147,148,149]. Moreover, lymphodepletion can increase the presentation of surface tumor antigens that can be presented to the infused T cells [150,151]. These benefits are visualized in Figure 3.

Specifically, CAR T cell therapy is augmented by lymphodepletion prior to CAR T cell transfer [152,153], with cyclophosphamide and fludarabine (alone or in combination) being the most common agents used to precondition the patient [154]. Additionally, in CD19-directed CARs, preconditioning patients with both cyclophosphamide and fludarabine improved T cell persistence [155]. We very recently reported that preconditioning with total body irradiation (TBI) or chemotherapy (cyclophosphamide/fludarabine alone or in combination) can differentially impact Th17 CD4 adoptive cellular therapy success, with some level of lymphodepletion needed for optimal efficacy [156]. Mechanistically, cytokines induced by lymphodepletion, such as MCP-1, are correlated with response; other cytokines, such as IL-7 and IL-15, are increased after lymphodepletion to aid T cell persistence and survival [153].

Importantly, though, patients with solid tumors may require differential methods of preconditioning compared to individuals diagnosed with hematologic malignancies prior to CAR T cell transfer. For example, in glioblastoma (GBM) patients, temozolomide increased CAR T cell persistence in the peripheral blood [157]. In another trial using an MPTK-CAR-T cell for mesothelin-positive tumors, cyclophosphamide and nab-paclitaxel with or without gemcitabine were used in the majority of patients [158]. Cyclophosphamide and fludarabine are still commonly used in the solid tumor context [159,160,161], yet they may not be the ideal candidates for each individual tumor type, and further investigation into these alternative regimens for preconditioning is still needed.

Lymphodepletion using either radiation or chemotherapy still has side effects for patients [154,162,163]—inspiring work by several groups to optimize CAR T cells to persist in the absence of lymphodepletion. This work includes strategies such as using CAR T cells that produce their own cytokines to augment persistence, such as with the engineered IL2 superkine (Super2) and IL33-producing CAR T cells [164] or IL-7 and CCL21-producing cells [165]. Another group identified STAT5b as a mediator of engraftment and used transient activation of this protein to improve transferred T cell persistence in lymphoreplete murine models [166]. These and other strategies may be able to decrease the need for lymphodepletion prior to cell transfer.

It is also possible that the type and level of lymphodepletion prior to CAR T cell therapy differ based on the patient’s diagnosed type of tumor. For example, CAR T cell therapy has shown some promise in treating intracranial brain tumors in the absence of lymphodepletion. In one case, multiple cycles of IL13Rα2-targeted CAR T cells were administered intracranially to a glioblastoma patient, leading to a complete response for 7.5 months before recurrence [167]. Another study involved intraventricular injections of CARv3-TEAM-E, targeting EGFRvIII and secreting T cell-engaging antibodies against wild-type EGFR, with mixed results: one patient had durable regression while the others had transient responses [168]. However, CAR T cell detection in cerebrospinal fluid (CSF) was limited between infusions. 

In a different trial, glioblastoma patients received CART-EGFR-IL13Rα2 cells targeting both EGFR and IL13Rα2 intrathecally through an intraventricular reservoir, leading to a transient regression of the tumor in all patients and early persistence of CAR T cells in the CSF [169]. Locoregional IL-13Rα2-targeted CAR T cell therapy, including intratumoral and intraventricular injections, has shown promise for high-grade gliomas, suggesting that locoregional delivery may mitigate the need for lymphodepletion [170]. It is possible that this success is in part due to locoregional delivery of CAR T cells to the brain, as a study using intravenous administration of anti-EGFRvIII CAR T cells combined with pembrolizumab showed no clinical efficacy in lymphoreplete glioblastoma patients [171]. Moreover, little clinical impact was observed when patients were given cyclophosphamide and fludarabine before intravenous anti-EGFRvIII CAR T trials for glioblastoma [172]. Without a clinical trial that formally tests if lymphodepletion augments CAR T cells against brain cancer, it will be hard to untangle the impact of lymphodepletion on patient outcomes.

Given the distinct biology of intracranial tumors, such as the blood–brain barrier and immunosuppressive microenvironment, it is not surprising that CAR T cells have been successful in lymphoreplete patients in this context [173]. However, the optimal lymphodepletion regimen for solid tumors and its impact on CAR T cell therapies remain a critical question. Lymphodepletion may be necessary to achieve sustained, long-term results and could have beneficial effects on CAR T cell function and efficacy.

### 2.7. Immune Checkpoint Blockade and CAR T Cells in Combination

Immune checkpoint blockade (ICB) has undoubtedly changed the landscape of cancer therapies, with PD-1, CTLA-4, and PD-1/LAG-3 therapies becoming FDA-approved for use in multiple malignancies, leading to improved survival for many patients [174,175]. ICB works by blocking inhibitory T cell interactions [176] and therefore has implications for CAR T cell therapy. ICB can reinvigorate CAR T cells after infusion, as the expansion of CD19-targeting CAR T cells was seen in a patient given PD-1 blockade therapy [177]. Data regarding the combination of CAR T cells and ICB have been reviewed [178], and pembrolizumab combined with CAR T cells was found to be safe in Phase I clinical trials for several tumor types [171,179,180]. In the initial studies combining pembrolizumab and CAR T therapy for glioblastoma, efficacy was not demonstrated [171]. Yet ultimately, combinations of CAR T cells with PD-1 and other ICB agents for different solid tumor types remain to be further investigated.

CAR T cells express inhibitory markers after infusion into patients [181], and PD-1 blockade improves CAR T expansion in certain subsets of patients [182]. Treatment of malignant pleural disease with mesothelin-directed CAR T products followed by pembrolizumab bolstered their expansion and increased disease response, including prolonged stable disease [179]. However, both the timing of administration and how it is administered are likely to impact outcomes. Additionally, other ICBs may synergize with CAR T therapy, with current FDA-approved Lag-3 and CTLA-4 checkpoint blockade therapies available. Combination therapies with both ICBs and other agents are potential strategies to further improve current CAR T therapies and need further investigation regarding their efficacy.

### 2.8. Vaccination

Another way to enhance CAR T cell expansion and persistence involves vaccine strategies. Cancer vaccines use tumor antigens combined with adjuvants to help stimulate the immune system. They have shown some success in patients with many different types of solid tumors [183,184,185,186,187]. Importantly, however, in addition to their potential use as single agents to induce immune responses against tumors, vaccines hold great promise as an additive to other immunotherapies such as immune checkpoint blockade and adoptive cellular therapy. If combined with CAR T cells in particular, vaccines have the potential to improve the persistence and durability of these ACT products for solid tumors.

Such vaccination strategies have been used successfully in preclinical models as well as in a clinical trial (NCT04503278). One group used an RNA vaccine strategy combined with claudin 6-targeting CAR T cells [188]. They administered liposomal claudin 6-encoding RNA (CLDN6-LPX) after CAR T administration and detected enhanced CAR T persistence [188] and tolerability in patients administered these agents in a Phase I clinical trial [189]. Another novel strategy for vaccination involved administering a CAR T ligand with amphiphilic properties, termed amph-ligand, that can traffic to the lymph node to encounter and activate CAR T cells [190]. Others used dendritic cells loaded with the CAR T target antigen as a vaccination strategy to enhance therapy efficacy [191].

Vaccination is therefore another way to enhance CAR T cell persistence. Unlike hematologic malignancies, where CAR T cells more rapidly encounter their antigen after infusion, solid tumors may benefit from vaccination and CAR T combination strategies to enhance their function, in turn bolstering their expansion and persistence until they reach their target. There are many considerations for developing the optimal vaccine for this context, which have been reviewed by others [192,193], and this topic deserves additional consideration.

## 3. Conclusions

**Figure 4:**
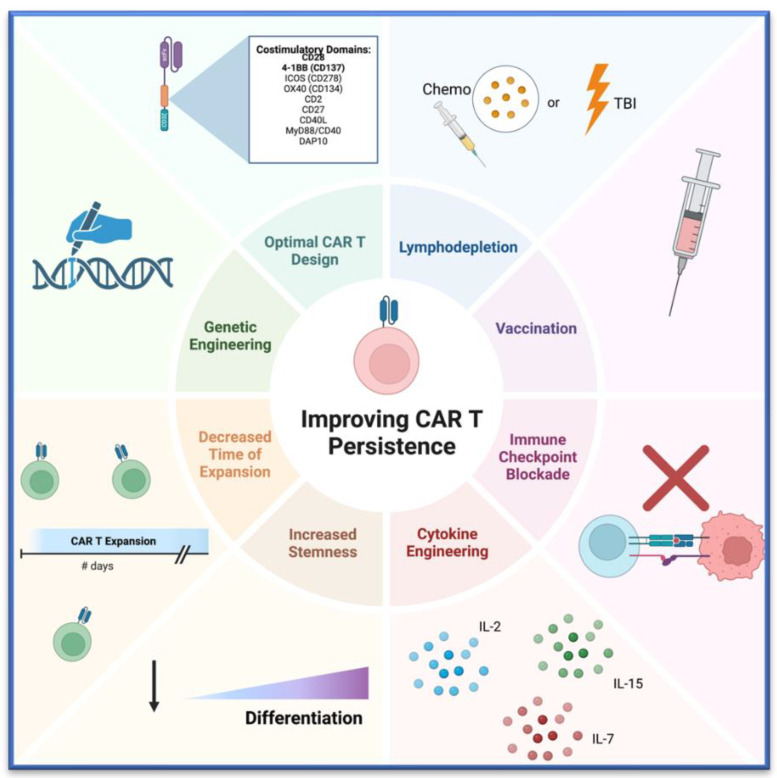
Depicted are the main strategies discussed throughout the review to improve CAR T cell persistence. Made using BioRender. TBI = total body irradiation.

The persistence of CAR T cell products is an important mediator of therapy success and is a factor that deserves attention to improve these therapies for use in solid tumors. There are many unique challenges with solid tumors that can impact persistence. This includes immunosuppressive microenvironments, trafficking to the tumor site, tumor heterogeneity, and other barriers. However, despite these many barriers, we remain optimistic about the potential of CAR T cell therapies to benefit patients with a variety of solid tumor types. Herein, we reviewed a plethora of strategies that can modulate CAR T cell persistence. In summary, optimizing CAR T cell therapy involves several key factors. We comprehensively capture these factors visually in Figure 4, and these include: designing CAR T cells with costimulatory domains to enhance persistence or genetic modifications to boost survival; selecting or enhancing memory-like CAR T cells through cytokines or pharmacologic agents; ensuring effective expansion processes; considering lymphodepletion to improve CAR T cell engraftment and survival; and employing vaccination and combination with ICBs to promote CAR T cell persistence.

Many strategies can be taken to improve CAR T cell success. The question of which path will ultimately be the best to pursue remains. We ourselves have focused on both enhancing T cell stemness for ACT and optimizing lymphodepletion regimens prior to transfer [88,156]. Yet, undoubtedly, the CAR T cell design is another essential factor that will impact the field. Identifying factors that can enhance T cell trafficking and survival in the tumor is one way we can tailor their potency, and this can be improved in a multitude of ways.

In the grand scheme of therapies, CAR T cells are still a relatively new form of medicine. Their success in several hematologic malignancies is to be celebrated. Bringing these therapies to patients with solid tumors will require active effort, but we are fortunate as a field to have many minds working on this goal. Here, we describe a multitude of potential solutions to help improve these therapies. We are encouraged by the preclinical and clinical studies outlined herein that show promise for enhancing CAR T cell persistence.

As this review was limited to describing strategies that impact the specific topic of CAR T cell persistence, there were several topics not thoroughly discussed that are still important considerations. This includes (1) identifying the target antigen of interest [194], (2) modulating the tumor microenvironment [131], (3) aiding in T cell infiltration into solid tumors [195], and other challenges. Some solutions to improve T cell trafficking are described, such as modulating chemokine signaling, and there are reviews that delve into this topic [196,197,198]. There are also tumor-intrinsic factors to consider, such as (1) antigen expression, (2) location of the tumor, (3) cellular composition, and more. Brain tumors were specifically highlighted for several unique factors, but each solid tumor type has various aspects to consider when designing the optimal CAR T therapy. Yet, despite these challenges, T cell therapies are emerging as viable strategies for patients with solid tumors.

In this review, we summarized the strategies that can most directly improve CAR T cell persistence and, therefore, efficacy in solid tumors. There are many strategies considered, and a focus on several of these will likely yield the most optimal results. While there is a need for continued improvement of CAR T cell therapies, the ongoing research and novel strategies described herein demonstrate the progress being made towards CAR T cell success in solid tumors.

## Figures and Tables

**Table 1:** FDA-approved T cell therapies for cancer. Accessed at https://www.fda.gov/vaccines-blood-biologics/cellular-gene-therapy-products/approved-cellular-and-gene-therapy-products on 5 August 2024.

Company Name	Generic Name	Target
ABECMA	idecabtagene vicleucel	BCMA
AMTAGVI	lifileucel	TIL for melanoma
BREYANZI	lisocabtagene maraleucel	CD19
CARVYKTI	ciltacabtagene autoleucel	BCMA
KYMRIAH	tisagenlecleucel	CD19
TECARTUS	brexucabtagene autoleucel	CD19
TECELRA	afamitresgene autoleucel	MAGE-A4
YESCARTA	axicabtagene ciloleucel	CD19
